# Aberrant expression of the MID1 protein in neurons of Huntington’s disease brain

**DOI:** 10.3389/fgene.2026.1753495

**Published:** 2026-03-11

**Authors:** Adriana Geraci, Annika Reisbitzer, Janina Gerhard, Sybille Krauß

**Affiliations:** Human- and Neurobiology, Institute of Biology, Department Chemistry and Biology, University of Siegen, Siegen, Germany

**Keywords:** Huntington’s disease, MID1, RNA-binding protein, translation regulation, ubiquitin ligase

## Abstract

Huntington’s disease (HD) is caused by a CAG repeat expansion mutation in the *Huntingtin* (*HTT*) gene that transcribes into mRNA and translates into a polyglutamine tract. The mutant *HTT* gene products drive pathological changes that result in neurodegeneration. The mutant CAG repeat RNA contributes to cellular dysfunction by aberrantly recruiting RNA-binding proteins. For example, the mutant *HTT* transcript associates with a protein complex containing the MID1 protein. This aberrant recruitment of the MID1 protein complex results in an increased translation of mutant *HTT*. MID1 expression is abnormally high in both the brains of HD mouse models and HD patients. However, the cell type in which MID1 is overexpressed in HD brains remains obscure. Here, we investigated the MID1 expression in different brain cell types of an HD mouse model. Therefore, we separated neurons, astrocytes and microglia via magnetic sorting and show that MID1 is overexpressed in neurons of an HD mouse model. Moreover, we stained MID1 in brain sections of HD mice via immunohistochemistry and observed MID1 overexpressing cells in cortex. This finding shows that MID1 is highly expressed in neurons–the most vulnerable cell type in HD–underlining its important role in the neurodegenerative process. This supports the concept of blocking the interaction between MID1 and mutant *HTT* mRNA to counteract mutant HTT translation as a promising therapeutic approach.

## 1 Introduction

Huntington’s disease (HD) is a fatal central nervous system disease that is caused by a CAG repeat expansion mutation in the *Huntingtin* (*HTT*) gene. This CAG repeat encodes an expanded polyglutamine tract in the mutant HTT protein (Huntington, 2003; The Huntington's Disease Collaborative Research Group, 1993). The mutant HTT protein aggregates in the patient’s brains, which is considered a pathological hallmark. In the last couple of years there is emerging evidence that in addition to the aggregation-prone neurotoxic polyglutamine protein the mutant CAG repeat RNA also causes cellular dysfunction [reviewed in (Nalavade et al., 2013; Schilling et al., 2016; Heinz et al., 2021a)]. The mutant CAG repeat RNA folds into an aberrant 3-dimensional hairpin structure that is not present in the normal *HTT* RNA (Sobczak et al., 2003; Krzysztof and Wlodzimierz, 2005; Kiliszek et al., 2010; de Mezer et al., 2011). This CAG repeat hairpin leads to a toxic gain-of-function by aberrantly recruiting RNA-binding proteins, that carry out abnormal functions in conjunction with the mutant CAG repeat RNA. One example of such an RNA-binding protein that gets trapped by the mutant *HTT* transcript is the Midline1 (MID1) protein. MID1 is a RING finger protein that contains several conserved domains: a RING finger, two B-Boxes, a coiled-coil domain, a Fibronectin type-III domain, and a B30.2/SPRY domain. All these domains can mediate protein-protein or nucleic acid-protein interactions. These include an E3 ubiquitin ligase activity mediated by the RING finger domain, the binding to the α4 regulatory subunit of protein phosphatase 2a to the B-Box1, the homo-dimerization of MID1 via the coiled-coil domain, or the association to microtubules via its C-terminal domains. MID1 plays a role in diverse cellular processes in healthy tissue but also under disease conditions (reviewed in (Winter et al., 2016)), including neurodegenerative diseases (Kickstein et al., 2010). One important function of MID1 is its ability to bind to mRNA and induce translation. This function is due to MID1’s ability to recruit a protein complex that contains various subunits of the eukaryotic translation initiation factor complex (eIF complex) as well as ribosomal subunits to its target mRNA (Matthes et al., 2018a). With respect to HD, the mutant *HTT* transcript aberrantly recruits the MID1 protein complex and this in turn drives translation of mutant *HTT* mRNA, resulting in aberrantly high levels of mutant HTT protein (Krauss et al., 2013).

Normally, the MID1 protein is ubiquitously expressed at low or medium levels [https://www.proteinatlas.org/ENSG00000101871-MID1/tissue (22.11.2025)]. Interestingly, the MID1 expression is aberrantly increased in HD brain tissue. This was observed both in an HD mouse model as well as in human HD brains (Heinz et al., 2021b). This makes the MID1 complex a promising pharmaceutical target to suppress the aberrant translation of mutant HTT in HD.

However, the exact brain cell type in which MID1 is overexpressed in HD remains unknown. Here we present data indicating that MID1 is aberrantly expressed in neurons in HD brain. This finding further strengthens the observation that MID1 is an important modifier in HD and targeting MID1 may represent a valuable treatment strategy.

## 2 Methods

### 2.1 Magnetic cell separation

The HdhQ150 mouse model (B6.129P2-Htt < tm2Detl>150J), which carries a mutant knock-in allele with 150 CAG repeats (encoding the HTT protein with 150Q repeats) was used (Lin et al., 2001). In total 14 mice (7 wild-type (wt) mice (4 male, 3 female) and seven heterozygous transgenic (tg) mice (4 male, 3 female) were analyzed. The procedures were approved by the state of North Rhine-Westphalia and were therefore in accordance with the German Animal Welfare Act (84-02.04.2017. A047). The mice were sacrificed at 15 weeks of age, and the brain tissue was dissociated using the Adult Brain Dissociation Kit (Miltenyi Biotech, 130-107-677) following the manufacturer’s instructions. Magnetic cell sorting (MACS) was then performed to separate neurons, microglia, and astrocytes. Therefore, the target cells were labeled with specific antibodies and coupled to magnetic beads, to retain specific cells when flowing the sample through the MACS columns, while the unlabeled cells flow through. Finally, by removing the magnetic field the target cells can be eluted. The Anti-ACSA-2 MicroBead Kit (130-097-678), the CD11b MicroBeads Kit (130-093-634), and the Neuron Isolation Kit (130-097-678) from Miltenyi Biotech were used for MACS. The procedure was carried out according to the manufacturer’s instructions.

### 2.2 Quantitative real-time PCR (qPCR)

Total RNA was isolated from magnetically sorted neurons, microglia, and astrocytes using the Monarch® Total RNA Miniprep Kit (NEB, T2010S). cDNA was synthesized with the TaqMan™ reverse transcription reagents kit (Applied Biosystems, N8080234), and real-time PCR was performed using the qPCRBIO SyGreen^®^ Mix (PCRBiosystems, PB20.11-01). Purification efficiency following MACS was validated by assessing canonical marker genes for astrocytes, microglia, and neurons (primers are listed in Supplementary Table S1). Relative quantification of *MID1* gene expression in all cell types was quantified using the ΔΔCt method. Statistical analysis was performed using GraphPad Prism 10.4.2 software (GraphPad Inc.). For the comparison of two groups, data were analyzed using the unpaired-Student’s *t*-test with Welch’s correction. Multiple groups were analyzed using one-way analysis of variance (ANOVA).

### 2.3 SH-SY5Y-Q68 cell line

The human neuroblastoma cell line SH-SY5Y, stably expressing GFP-tagged mutant HTT exon1 with 68 CAG repeats under a doxycycline-inducible promoter (Schilling et al., 2019), was cultured in Dulbecco’s modified Eagle’s medium F-12, GlutaMAX™ supplement (DMEM/F-12; Gibco™, 10565018) with 10% tetracycline free, heat-inactivated fetal bovine serum (FBS; PAN-Biotech, P30-3602). Cells were maintained at 37 °C and 8% CO_2_ in humidified air and sub-cultured upon reaching 70%-80% confluency.

### 2.4 Time course of differentiation and MID1 knock-down

On day −2, cells were seeded at a density of 1.2 × 10^5^ cells in DMEM/F12 supplemented with 10% tetracycline-free, heat-inactivated FBS in standard 12-well plates (Sarstedt, 83.3921). For immunofluorescence, 3.6 × 10^5^ cells were seeded onto coverslips coated with 0.2 mg/mL Poly-D-Lysine (Sigma, P-7886) and 2 μg/mL Laminin (Sigma, L2020) in 6-well plates (Sarstedt, 83.3920.005). MID1 knock-down was performed on day −1 using Lipofectamine™ RNAiMAX Transfection Reagent (Invitrogen™, 13778075) and a pool of five MID1 siRNAs (Supplementary Table S3). On day 0, cells were washed with PBS, and neuronal differentiation was induced by treatment with 10 µM retinoic acid (RA; Sigma Aldrich, R2625) in DMEM/F-12 containing tetracycline-free, heat-inactivated 2% FBS. On days 3 and 6, cells were washed with PBS, and the differentiation treatment was repeated with the addition of 50 ng/mL brain-derived neurotrophic factor (BDNF; Sigma, B3795) and a reduction of FBS to 1%. On day 6, *HTT*exon1-Q68 expression was induced by adding 1 μg/mL doxycycline (Figure 3A). Cells were lysed on Day 0, 3, 6 and 8 in ice-cold lysis buffer (50 mM TRIS, 100 mM NaCl, 5 mM MgCl2, 1% NP-40, 1 mm EDTA, 50 units/μL benzonase, complete protease inhibitor (Thermo Fisher, A32963), pH 8.8) and stored at −20 °C until required.

### 2.5 Immunofluorescence of neuronal differentiated cells

Coverslips were washed with PEM buffer (0.1 M PIPES pH 7.2, 2 mM MgCl_2_, 0.5 mM EGTA) and fixed in 4% paraformaldehyde (PFA) for 10 min at room temperature. Following fixation, cells were washed with PBS, permeabilized using 1% Triton X-100 for 10 min at room temperature, and washed again with PBS. To prevent non-specific antibody binding, samples were blocked in 1% BSA for 1 h at room temperature. Primary antibodies were diluted 1:100 in 3% BSA in PBS and incubated for 1 h at room temperature. After washing with PBS, samples were incubated with the secondary antibody at a 1:1000 dilution for 1 h at room temperature. Antibodies are listed in Supplementary Table S2. Following a final PBS wash, coverslips were mounted in DAPI-containing Fluoroshield™ medium (Sigma Aldrich, F6057). Images were captured using the LSM900 confocal fluorescence microscope (ZEISS) with a 63× oil-immersion objective.

### 2.6 Western blot

Protein samples were resuspended in 2x Magic Mix (48% Urea, 15 mM Tris-HCl pH 7.5, 8.7% Glycerol, 1% SDS, 0.004% Bromophenol blue, 143 mM Mercaptoethanol) and denatured at 95 °C for 10 min. Samples were run on NuPAGE™ 4%-12% Bis-Tris Gel (NP0322BOX, Thermo Fisher) in 1x NuPAGE MOPS SDS running buffer (NP0001, Thermo Fisher). Western blot was performed using semi-dry blotting on nitrocellulose membranes (PB9320, Thermo Fisher). The membranes were incubated with the antibodies listed in Supplementary Table S2. For quantification, band intensities were measured with the iBright analysis software (Invitrogen), and data normalization of MID1 signals was performed using the band intensity of ACTB as a loading control.

### 2.7 Immunohistochemistry (IHC)

#### 2.7.1 IHC double staining

15 weeks old wt and heterozygous tg mice (male) were euthanized by cervical dislocation, and brains were dissected. Tissue was shock-frozen at −80 °C and embedded in Tissue-Tek O.C.T. compound. Coronal cryosections were cut at −20 °C using a CryoStar NX70 cryostat (Thermo Fisher Scientific). Sections were 10 µm thick. For immunohistochemical analyses, the cryosections were fixed in ice-cold methanol for 10 min and air-dried. Immunohistochemical staining was performed using the NeoStain Poly DS Kit–for Mouse and Rabbit antibody on Mouse tissue (Emerald/Permanent Red) (NeoBiotech, NB-23-00096-1) according to the manufacturer’s instructions with minor deviations. Slides were incubated overnight at 4 °C in the primary antibodies (Supplementary Table S2). Images were captured between 10× and 100× magnification using an inverted fluorescence-phase contrast microscope (Keyence, BZ-X810).

#### 2.7.2 IHC triple immunofluorescence staining

Cryosections were rehydrated in ice-cold PEM buffer, fixed in ice-cold PFA for 10 min, and washed 3 times with PEM buffer. Antigen retrieval was carried out using 1% SDS in PEM for 3 min. Following PBS washes, tissue was permeabilized with 0.3% Triton X-100 for 10 min and blocked with 10% FBS for 60 min at room temperature to prevent nonspecific binding. For immunofluorescence staining, sections were incubated overnight at 4 °C with primary antibodies (Supplementary Table S2). After washing with PBS, sections were incubated with the corresponding fluorophore-conjugated secondary antibodies at 4 °C for an additional night. Following PBS washes, the slides were mounted in DAPI-containing Fluoroshield™ medium (Sigma Aldrich, F6057). Images were captured using the LSM900 confocal fluorescence microscope (ZEISS) with either a 20x or a 63× oil-immersion objective.

## 3 Results

### 3.1 *MID1* mRNA expression is aberrantly increased in HD-neurons

**FIGURE 1:**
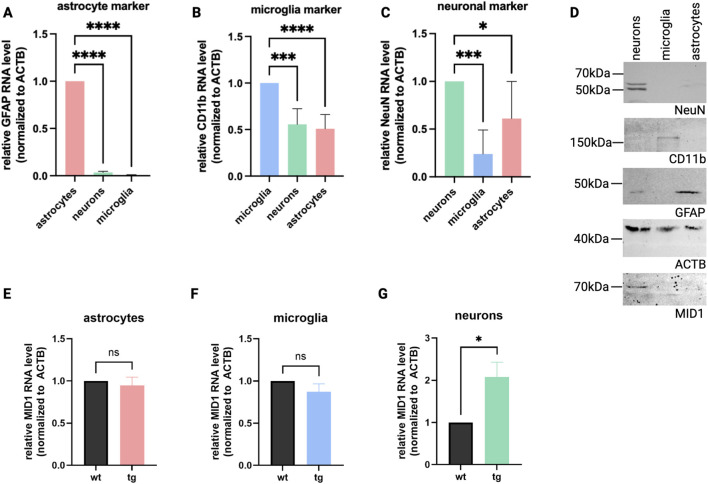
*MID1* mRNA expression is aberrantly increased in HD-neurons. The *MID1* expression was quantified in distinct cell populations isolated by MACS from the cortex of 15-week-old HdhQ150 knock-in mice (tg) and age-matched wild-type littermates (wt). The success of separation was quantified by qPCR analysis detecting GFAP as marker for astrocytes **(A)**, CD11b as marker for microglia **(B)**, and NeuN as neuronal marker **(C)**. **(D)** The marker proteins GFAP, NeuN, and CD11b were also detected on a Western blot of pooled samples from the tg animals (n = 7). MID1 was detected on the same blots. *MID1* qPCR analysis revealed no statistically significant differences in **(E)** astrocytes (*p* = 0.595) and **(F)** microglia (*p* = 0.1966). **(G)** In neurons a significant upregulation of *MID1* expression was detected (*p = 0.0104). *MID1* levels were normalized to *ACTB*. Columns represent mean values ± SEM, *p*-values were calculated using an unpaired t-test with Welch’s correction (n_wt_ = 7, n_tg_ = 7).

Our previous studies showed that *MID1* expression is aberrantly increased in brain tissue of an HD mouse model HdhQ150 at the early age of 10–15 weeks (young adults compared to humans), a state characterized by a complex change in neuronal activity pattern and hyperactive neurons that is accompanied by behavioral changes (Heinz et al., 2021b). Thus, to identify the cell type in which *MID1* is overexpressed we used 15-week-old mice for our analysis. We first isolated different cell types of the animals’ brains by magnetic separation from tg and wt mice. The successful separation was shown by detection of neuronal, astrocytic, and microglial markers (Figure 1). From these different cell types, we isolated total RNA and analyzed the *MID1* expression via qPCR. We detected a significant upregulation of *MID1* in neurons from HD animals, while no significant difference between wt and tg animals was detectable in astrocytes or microglia (Figure 1).

### 3.2 MID1 protein level is aberrantly increased in HD-neurons

**FIGURE 2:**
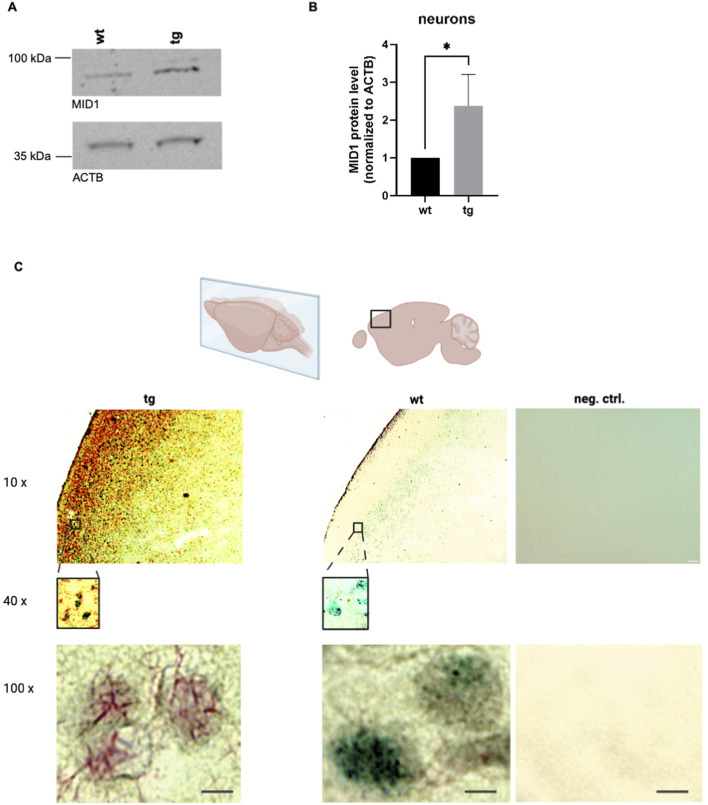
MID1 protein level is aberrantly increased in HD neurons. **(A)** Western blot analysis of neurons isolated from wt and tg mice were performed detecting the MID1 protein or ß-actin (ACTB) as loading control. A representative image of *n* = 5 experiments is shown. **(B)** Quantification of the bands on western blots described in **(A)** is shown. Columns represent mean values ±SEM of relative MID1 protein level, normalized to ACTB. Unpaired t-test was performed to indicate significant efficiency. *p = 0.0432. **(C)** Immunohistochemistry (IHC) using a dual polymer system. The MID1 protein is detected using an anti-MID1 antibody and a permanent red substrate (red), while the neuronal marker NeuN is detected using an anti-NeuN antibody and an emerald green substrate (green). Left: IHC staining of MID1 and NeuN in cortical tissue of tg mice. Middle: IHC staining in wt animals. Right: Negative control staining without primary antibodies. Scale bar 3 µm. Schematic of mouse brain and selected region was drawn using biorender.com.

To validate the observed increase of *MID1* expression in neurons on protein level, neurons were isolated from wt and tg animals of our HD mouse model as described above and analyzed on western blots. In line with the above-mentioned results, we also detected upregulated MID1 protein levels of HD neurons (Figure 2). Moreover, immunohistochemistry staining of MID1 in coronal sections of these animals further supported that MID1 is highly expressed in neurons of HD animals, since MID1-positive cells co-stained for the neuronal marker NeuN (Figure 2). Co-staining of MID1 and HTT showed that MID1-positive cells contain HTT inclusions in HD animals, while HTT staining in control animals was much weaker (Supplementary Figure S1A). Similarly, a triple immuno-staining of MID1, HTT, and a neuronal marker (β III tubulin) showed that high levels of MID1 and HTT co-incite in neurons (Supplementary Figure S1B). While the HTT protein signal seems stronger in the HD animals compared to controls, the HTT mRNA level did not differ significantly (Supplementary Figure S1C). This is in line with the known translation-promoting function of MID1 (Krauss et al., 2013).

**FIGURE 3:**
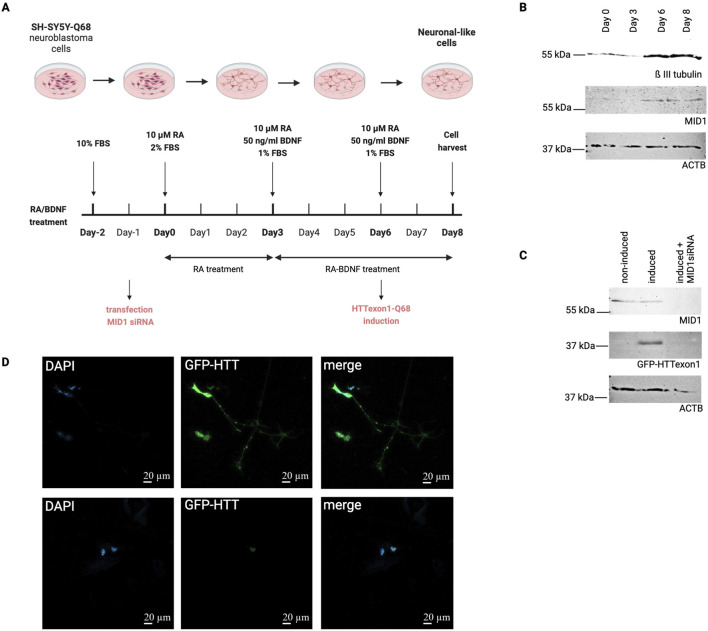
MID1 is upregulated in a neuronal HD cell model. **(A)** Experimental timeline of neuronal differentiation protocol. SH-SY5Y-Q68 cells are seeded on day −2 followed by MID1 siRNA transfection on day −1. Neuronal differentiation was initiated on day 0 with 10 µM RA. On days 3 and 6, cells received RA plus 50 ng/mL BDNF and reduced serum (1% FBS). Expression of GFP-tagged *HTT*exon1-Q68 was induced on day 6. Schematic was drawn using biorender.com. **(B)** Western blot analysis indicating success of neuronal differentiation by increased level of the neuronal marker β III tubulin on days 6 and 8. Detection of MID1 on the same blots reveals increased expression upon differentiation. **(C)** Western blot analysis of differentiated cells in day 8 in the presence (right lane) or absence (middle lane) of MID1 specific siRNAs. A marked decrease of GFP-HTT is detected in cells that underwent MID1 knockdown. A sample of cells that were not induced for expression of GFP-tagged *HTT*exon1-Q68 was loaded as negative control (left lane). **(D)** Fluorescence microscopy of differentiated SH-SY5Y-Q68 cells expressing GFP-tagged *HTT*exon1-Q68 in the presence (lower panel) or absence (upper panel) of MID1siRNA. DAPI was stained to visualize nuclei.

To further validate the increased expression of MID1 in HD neuronal cells, we made use of a previously established cell line SH-SY5Y-based model stably expressing GFP-tagged mutant HTT exon1 under an inducible promoter (Schilling et al., 2019). We induced neuronal differentiation of these cells by adding RA and BDNF to the culture medium (Figure 3A). As expected, the MID1 protein level in the undifferentiated cells was weak, while it increased upon neuronal differentiation (Figure 3B). Upon induction of mutant HTT exon1 expression, a clear signal for GFP-HTT was detectable. In line with our previous studies on the translation-promoting function of MID1 (Krauss et al., 2013), siRNA-mediated knock-down markedly decreased the GFP-HTT level (Figure 3C). A similar effect was visible in immunofluorescence imaging of these cells (Figure 3D).

Together our data show that *MID1* expression is increased in cortical neurons of HD mice, while no overexpression of *MID1* is observed in astrocytes or microglia.

## 4 Discussion

RNA metabolism, including its processing and translation, is a tightly regulated cell-type-specific process. Aberrant RNA-protein interactions leading to deregulated RNA metabolism may lead to a plethora of diseases. One example of a disease-relevant aberrant RNA-protein interaction is the recruitment of the MID1 protein complex to mutant *HTT* mRNA (Krauss et al., 2013). The MID1 protein is known to promote translation of its target mRNAs (Krauss et al., 2013; Griesche et al., 2016; Hettich et al., 2014; Kohler et al., 2014). Thus, in HD cell models, the sequestration of MID1 to mutant *HTT* RNA results in an increased translation rate of the mutant HTT protein (Krauss et al., 2013). Moreover, our previous studies showed that MID1 is aberrantly high expressed in HD brain (Heinz et al., 2021b).

However, the cell type of MID1 overexpressing cells in the HD brain has not been studied thus far. Our data show that this aberrant MID1-expression takes place in neurons, the cell type that degenerates in the course of disease.

We show here that MID1 is overexpressed in neurons of HD animals at an age of approximately 15 weeks. Previously MID1 expression was mainly investigated in embryos or very young individuals: In mice MID1 is expressed in embryos between E8.5-14.5 and in newborn animals. In humans MID1 is expressed in different tissues depending on age, including a peak of expression in 5-7 weeks old embryos in the central nervous system (reviewed in (Winter et al., 2016)). While MID1 is normally ubiquitously expressed at low or medium levels [https://www.proteinatlas.org/ENSG00000101871-MID1/tissue (22.11.2025)] in adult tissue, our previous (Heinz et al., 2021b) and current data show a significantly upregulated expression of MID1 under disease-conditions in HD. Interestingly, we detect overexpression of MID1 already at a relatively young age of our experimental animals at an age of approximately 15 weeks (young adults compared to humans), indicating that MID1 overexpression occurs as an early event in the course of the disease. Since MID1 is known to regulate patterning in the developing human brain (Frank et al., 2024), future studies should aim at investigating the effect and function of MID1 in HD brain also at earlier stages of brain development.

While our data presented here identify neurons as the cell type that expresses MID1 at abnormally high levels, the underlying mechanism that induces MID1 expression in HD remains obscure and should be investigated in future studies. Known regulators of MID1 expression include TRAIL (Tumour necrosis factor-related apoptosis-inducing ligand) (Collison et al., 2015), DREAM (Downstream regulatory element antagonist modulator) (Dierssen et al., 2012), and microRNAs miR-19, miR-340, miR-374 and miR-542 (Unterbruner et al., 2018).

Our data show that MID1 expression is aberrantly increased in HD brains in neurons, the most vulnerable cell type. This together with the stimulating effect of MID1 on mutant HTT synthesis makes MID1 a promising therapeutic target to halt HD disease progression. Indeed, different experimental approaches blocking the interaction between mutant *HTT* mRNA and MID1 gave promising results. For example, blocking the CAG repeat RNA by the RNA-binder furamidine interferes with the recruitment of MID1 to mutant *HTT* RNA and suppresses MID1-dependent translation (Matthes et al., 2018b).

In another study targeting the MID1 protein complex with the antidiabetic drug metformin also resulted in decreased mutant *HTT* translation. In detail metformin affects the binding of MID1 to its interacting proteins α4 and PP2Ac, which is crucial for its translation regulator function (Kickstein et al., 2010). Thereby metformin blocks aberrant HTT translation and also restores early functional changes and behavioural aberrations in an HD mouse model (Arnoux et al., 2018). Similarly, another study applying a peptide that disrupts the interaction between MID1 and α4 showed decreased HTT protein translation in cultures of neurons derived from HD mice (Monteiro et al., 2018). Together, all these data support that MID1 is a valuable drug target. Future studies should further develop and refine MID1-inhibitors as potential drugs for treating HD. Interestingly, MID1 also binds to expanded CAG repeat RNA in models of other CAG repeat expansion diseases (Griesche et al., 2016). Thus, a small molecule targeting the MID1-CAG-repeat RNA binding may be useful in the context of other CAG repeat expansion diseases as well.

Of note, MID1 is not the only RNA-binding protein that is abnormally recruited to the mutant *HTT* RNA. Various studies have identified other RNA-binding proteins that interact with the expanded CAG repeat RNA, leading to cellular dysfunction. These include, for example, nucleolin, which binds to expanded *HTT* RNA and is associated with nucleolar dysfunction, altered ribosome biogenesis, and impaired proteostasis (Tsoi et al., 2012; An et al., 2022; Sonmez et al., 2021). Another example would be splice factors or translation regulators such as MID1 (Krauss et al., 2013; Schilling et al., 2019).

A comparison of the proteins identified by Schilling et al. (2019) that bind the mutant *HTT* RNA depending on the length of the CAG repeat with the MID1-interacting proteins described by Matthes et al. (2018a) shows that various proteins interact with both MID1 and the mutant *HTT* RNA (Supplementary Table S4). Therefore, it is possible that altered MID1 expression influences the binding of these proteins to *HTT* RNA. Although this still needs to be investigated in future studies, it is possible that increased MID1 levels may affect the recruitment of cytosolic CAG binders such as translation factors. This is in line with our previous studies showing that depletion of MID1 leads to the “detachment” of S6K (Krauss et al., 2013). However, nuclear proteins are unlikely to be affected by increased expression of MID1, as they bind to a different subcellular pool of HTT RNA.

Taken together, our data show an aberrant upregulation of MID1 in HD neurons, but not in glia cells, which affects the neuronal translational machinery. Besides such RNA-mediated mechanisms of cellular dysfunction also other pathomechanisms contribute to HD disease development, for example neuroinflammation. Interestingly, MID1 responds to inflammatory reaction and is upregulated in inflammatory diseases of the respiratory tract and esophagus (Collison et al., 2015; Collison et al., 2013; Collison et al., 2020). Future work should address if HD neurons in which MID1 is upregulated may show signs of neuroinflammation and if neuroinflammation may be a trigger for this upregulation of MID1 expression. Moreover, neuroinflammation in the HD brain is marked by reactive microglia and astrocytes. Recent evidence suggests that their activation promotes chronic inflammation and contributes to neuronal dysfunction and death. However, these glial cells can also exert protective functions that help limit tissue damage (Palpagama et al., 2019). A better understanding of their dual roles as well as their crosstalk with neurons may enable the development of targeted therapeutic strategies for HD. In this aspect it will be interesting to address in future studies, if RNA-mediated mechanisms of cellular dysfunction in neurons, like upregulation of MID1, are also connected to glial cell disturbance.

## Data Availability

The original contributions presented in the study are included in the article/Supplementary Material, further inquiries can be directed to the corresponding author.
