# Supplementary material for: Aberrant expression of the MID1 protein in neurons of Huntington’s disease brain
Source: Front Genet. 2026 Mar 11;17:1753495. doi: 10.3389/fgene.2026.1753495 (PMC13012708; doi:10.3389/fgene.2026.1753495)
Supplement: Supplementary file 1 [file Supplementaryfile1.docx]

Supplementary Material

# Supplementary Tables and Figures

## Supplementary Table 1

Table 1: List of quantitative PCR primers

| **Gene names** | **Forward sequence 5’ 🡪 3’** | **Reverse sequence 5’ 🡪 3’** | |
| --- | --- | --- | --- |
| *ACTB* | TACCACAGGCATTGTGATG | TTTGATGTCACGCACGATTT |  |
| *GFAP* | AACCGCATCACCATTCCTG | GCATCTCCACAGTCTTTACCA |  |
| *HTT* | ATGGCAACCCTGGAAAAGC | CTGCTGTTGCTGCTGAAACG |  |
| *ITGAM* (CD11b) | CCACAGTTCACACTTCTTTCAG | TGTCCAGATTGAAGCCATGA |  |
| *MID1* | GAAGACCAACAGTCAGCCGT | ACAGTCAGGTTGTCGTGGGA |  |
| *RBFOX3* (NeuN) | ATACATTTCGAGCTGCACCA | CCTCCATAAATCTCAGCACCA |  |

## Supplementary Table 2:

Table 2: List of primary antibodies used for western blotting, immunofluorescence and immunohistochemistry

| **Primary antibody** | **Species** | **Company** | **Cat number** | **Dilution** |
| --- | --- | --- | --- | --- |
| anti-beta-Actin (13E5) HRP | rabbit | Cell Signaling Technology | #5125 | 1:100 WB |
| anti-beta III tubulin | rabbit | Abcam | ab18207 | 1:1000 WB |
| anti-beta III tubulin | chicken | Abcam | ab41489 | 1:1500 IF |
| anti-CD11b/ITGAM (E4K8C) | rabbit | Cell Signaling Technology | #93169 | 1:1000 WB |
| anti-GFAP (2E1) | mouse | Santa Cruz  Biotechnology | sc-33673 | 1:1000 WB |
| anti-GFP HRP | goat | Abcam | ab6663 | 1:500 WB |
| anti-HTT, clone mEM48 | mouse | Merck Millipore | MAB5374 | 1:250 IHC 1:50 IF |
| anti-MID1 | rabbit | Described in [1] |  | 1:500 WB 1:100 IHC |
| anti-MID1 | rabbit | Abcam | ab70770 | 1:100 IF |
| anti-NeuN, clone A60 | mouse | Merck Millipore | MAB377 | 1:1000 WB 1:100 IHC |
| **Secondary antibody** | **Species** | **Company** | **Cat number** | **Dilution** |
| goat-anti-mouse IgG H&L (Alexa Fluor® 647) | goat | Abcam | ab150115 | 1:200 IF |
| goat-anti-rabbit IgG  (Alexa Fluor® 555) | goat | Abcam | ab150078 | 1:200 IF |
| goat-anti-chicken IgY H&L (Alexa Fluor® 488) | goat | Thermo Fisher Scientific | A-11039 | 1:200 IF |
| mouse anti-rabbit IgG-HRP | mouse | Santa Cruz Biotechnology | sc-2357 | 1:1000 WB |

## Supplementary Table 3:

Table 3: List of MID1 siRNA-mediated knock-down sequences

| **Gene name** | **Sequence 5’ 🡪 3’**  **Company**  **Cat number** | **Concentration** |
| --- | --- | --- |
| *MID1* | AATTGACAGAGGAGTGTGATC  CACCGCAUCCUAGUAUCACACTT | 0.32 nM |
|  | CAGGAUUACAACUUUUAGGAATT |  |
|  | CAGGAUUACAACUUUUAGGAATT |  |
|  | AAGGTGATGAGGCTTCGCAAA |  |
|  | TAGAACGTGATGAGTCATCAT |  |

##

## Supplementary Table 4:

Table 4: Proteins binding *HTT*-RNA in a CAG-repeat length dependent manner

| **Proteins binding *HTT*-RNA in a CAG-repeat length dependent manner identified by Schilling et al. [2]** | | **Proteins in MID1-interactome identified by Matthes et al. [3]** |
| --- | --- | --- |
| **Gene names** | **Protein names** |  |
| *RBM25* | RNA-binding protein 25 | **x** |
| *SF3A3* | Splicing factor 3A subunit 3 |  |
| *DHX15* | Putative pre-mRNA-splicing factor ATP-dependent RNA helicase DHX15 | **x** |
| *SF3B3* | Splicing factor 3B subunit 3 | **x** |
| *SF3B2* | Splicing factor 3B subunit 2 |  |
| *SNRNP200* | U5 small nuclear ribonucleoprotein 200 kDa helicase | **x** |
| *SF3B1* | Splicing factor 3B subunit 1 | **x** |
| *HNRNPC* | Heterogeneous nuclear ribonucleoproteins C1/C2 | **x** |
| *DDX46* | Probable ATP-dependent RNA helicase DDX46 |  |
| *U2AF2* | Splicing factor U2AF 65 kDa subunit | **x** |
| *SNRNP40* | U5 small nuclear ribonucleoprotein 40 kDa protein | **x** |
| *EIF4A3* | Eukaryotic initiation factor 4A-III | **x** |
| *PUF60* | Poly(U)-binding-splicing factor PUF60 | **x** |
| *SF3B4* | Splicing factor 3B subunit 4 |  |
| *SNRPD1* | Small nuclear ribonucleoprotein Sm D1 | **x** |
| *SF3A1* | Splicing factor 3A subunit 1 |  |
| *PRPF8* | Pre-mRNA-processing-splicing factor 8 | **x** |
| *PRPF31* | U4/U6 small nuclear ribonucleoprotein Prp31 | **x** |
| *EFTUD2* | 116 kDa U5 small nuclear ribonucleoprotein component | **x** |
| *PRPF19* | Pre-mRNA-processing factor 19 | **x** |
| *PRPF40A* | Pre-mRNA-processing factor 40 homolog A |  |
| *SRSF3* | Serine/arginine-rich splicing factor 3 | **x** |
| *RBM39* | RNA-binding protein 39 |  |
| *SON* | Protein SON |  |
| *SRSF1* | Serine/arginine-rich splicing factor 1 | **x** |
| *BCLAF1* | Bcl-2-associated transcription factor 1 |  |
| *SRRM2* | Serine/arginine repetitive matrix protein 2 |  |
| *RPS27* | 40S ribosomal protein S27;40S ribosomal protein S27-like | **x** |
| *U2SURP* | U2 snRNP-associated SURP motif-containing protein | **x** |
| *RALY* | RNA-binding protein Raly |  |
| *HNRNPUL2* | Heterogeneous nuclear ribonucleoprotein U-like protein 2 |  |
| *DHX36* | Probable ATP-dependent RNA helicase DHX36 |  |
| *ZC3HAV1* | Zinc finger CCCH-type antiviral protein 1 | **x** |
| *SRSF6* | Serine/arginine-rich splicing factor 6 |  |
| *TRA2B* | Transformer-2 protein homolog beta |  |
| *PNN* | Pinin |  |

## Supplementary Figure 1


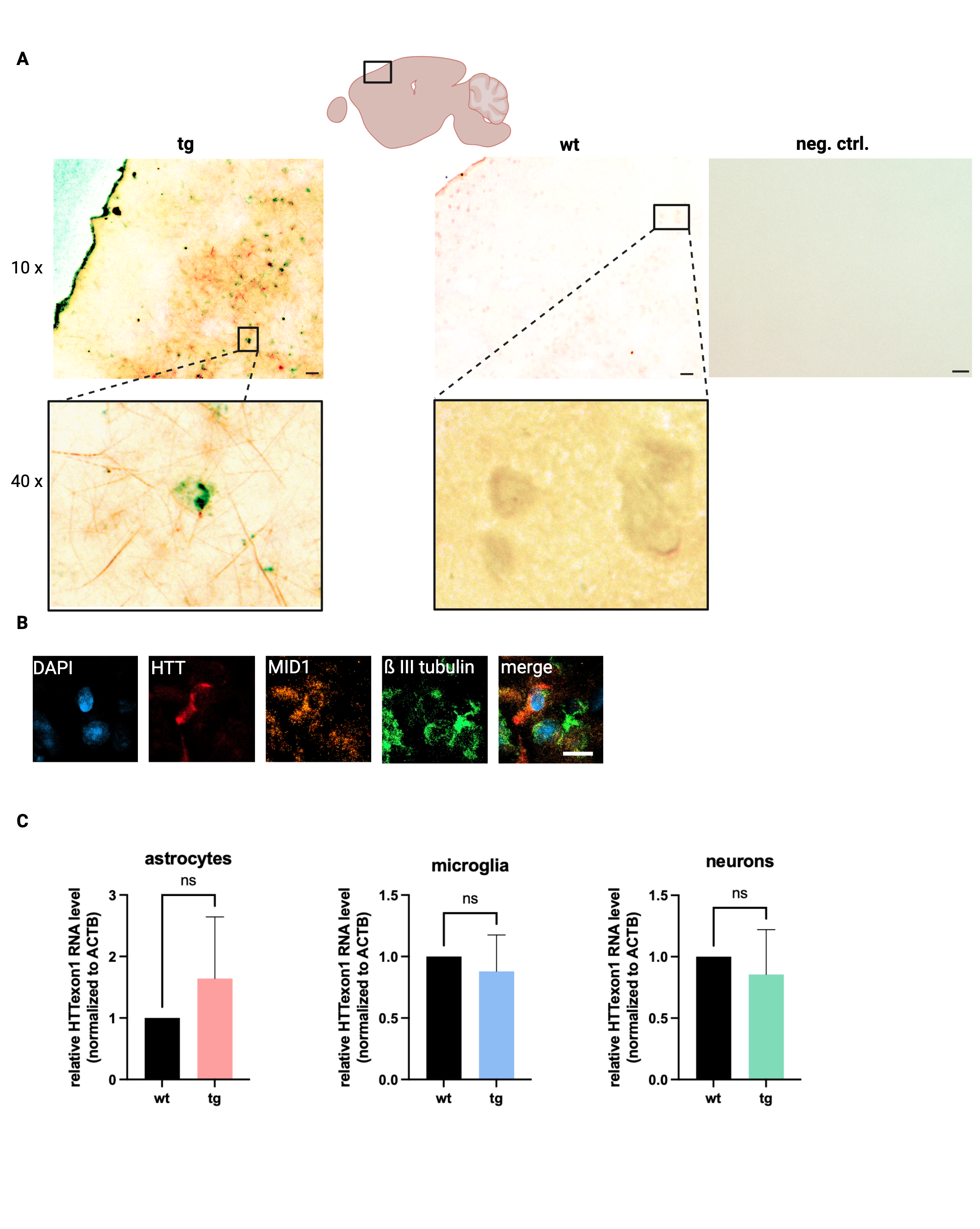


**Supplementary Figure 1:** (A) Double staining of HTT-MID1 in cortical tissue. The MID1 protein is detected using an anti-MID1 antibody and a permanent red substrate (red), while HTT is detected using an anti-HTT antibody and an emerald green substrate (green). Left: IHC staining of MID1 and HTT in cortical tissue of tg mice. Middle: IHC staining in wt animals. Right: Negative control staining without primary antibodies. Scale bar 3 µm. Schematic of mouse brain and selected region was drawn using biorender.com. (B) Triple immunofluorescence staining for HTT (red), MID1 (orange), and β III tubulin (green) in cortical tissue. Nuclei were counterstained with DAPI (blue). Images were captured using the LSM 900 confocal fluorescence microscope with a 63× oil-immersion objective. Scale bar 10 µm. (C) *HTT* mRNA level did not differ significantly in neurons, astrocytes, and microglia. HTT expression was quantified in distinct cell types revealing no statistically significant difference in astrocytes (*p* = 0.2896), microglia (*p* = 0.4739) and neurons (*p* = 0.4871). *HTT* levels were normalized to ACTB. Columns represent mean values ± SEM, *p*-values were calculated using an unpaired t-test with Welch’s correction (n_wt_ = 4, n_tg_ = 4).

[1] S. Schweiger, F. Matthes, K. Posey, E. Kickstein, S. Weber, M.M. Hettich, S. Pfurtscheller, D. Ehninger, R. Schneider, and S. Krauss, Resveratrol induces dephosphorylation of Tau by interfering with the MID1-PP2A complex. Sci Rep 7 (2017) 13753.

[2] J. Schilling, M. Broemer, I. Atanassov, Y. Duernberger, I. Vorberg, C. Dieterich, A. Dagane, G. Dittmar, E. Wanker, W. van Roon-Mom, J. Winter, and S. Krauss, Deregulated Splicing Is a Major Mechanism of RNA-Induced Toxicity in Huntington's Disease. J Mol Biol (2019).

[3] F. Matthes, M.M. Hettich, J. Schilling, D. Flores-Dominguez, N. Blank, T. Wiglenda, A. Buntru, H. Wolf, S. Weber, I. Vorberg, A. Dagane, G. Dittmar, E. Wanker, D. Ehninger, and S. Krauss, Inhibition of the MID1 protein complex: a novel approach targeting APP protein synthesis. Cell Death Discovery 4 (2018).
